# *Pseudomonas yongnianensis* sp. nov., Isolated from the Rhizosphere Soil of Wheat in a High-Yield Farmland

**DOI:** 10.3390/microorganisms14102363

**Published:** 2026-10-15

**Authors:** Yidan Yan, Daseul Lee, Jun Heo, Seung-Beom Hong, Wei Chen, Yuanhong Wang, Hailei Wei

**Affiliations:** 1 State Key Laboratory of Efficient Utilization of Arable Land in China, Institute of Agricultural Resources and Regional Planning, Chinese Academy of Agricultural Sciences, Beijing 100081, China; 821012410001@caas.cn (Y.Y.); weihailei@caas.cn (H.W.); 2 Agricultural Microbiology Division, National Institute of Agricultural Sciences, Rural Development Administration, Wanju 55365, Republic of Korea; daseul0213@korea.kr (D.L.); bioheojun@korea.kr (J.H.); funguy@korea.kr (S.-B.H.); 3 College of Horticulture and Landscape Architecture, Tianjin Agricultural University, Tianjin 300392, China

**Keywords:** *Pseudomonas yongnianensis* sp. nov., novel species, high-yield wheat field, carotenoid, type III secretion system

## Abstract

A novel *Pseudomonas* strain, designated S9268^T^, was isolated from the rhizosphere soil of wheat in Yongnian District, Handan City, Hebei Province, PR China. Cells were Gram-stain-negative, aerobic, rod-shaped, and motile by means of multiple polar flagella. Phylogenetic analyses based on 16S rRNA gene and multilocus sequence analysis (MLSA) of four housekeeping genes (*16S rRNA* gene, *gyrB*, *rpoB*, and *rpoD*) placed strain S9268^T^ within the genus *Pseudomonas* but as a distinct lineage separated from its closest relatives, *Pseudomonas asturiensis* LMG 26898^T^, *Pseudomonas avellanae* LMG 21662^T^, and *Pseudomonas lijiangensis* JCM 35177^T^. The average nucleotide identity (ANI) and digital DNA–DNA hybridization (dDDH) values between strain S9268^T^ and three reference strains ranged from 83.1% to 83.5% and from 24.9% to 25.6%, respectively, which were below the recommended thresholds for prokaryotic species delineation. The strain can grow at 4–37 °C (optimum, 28 °C), at pH 6.0–9.0 (optimum, pH 7.0), and with 1–4% (*w*/*v*) NaCl (optimum, 1%). The major cellular fatty acids (>10% of the total) were C_16:0_, summed feature 3 (C_16:1_ ω7c and/or C_16:1_ ω6c), summed feature 8 (C_18:1_ ω7c and/or C_18:1_ ω6c), and C_17:0_ cyclo. The polar lipid profile consisted of phosphatidylethanolamine, diphosphatidylglycerol, phosphatidylglycerol, three unidentified aminophospholipids, an unidentified phosphoglycolipid, and an unidentified polar lipid. The predominant respiratory quinone was ubiquinone Q-9. The draft genome of strain S9268^T^ was 5,250,102 bp in length with a DNA G+C content of 61.2 mol%. Genome analysis revealed T3SS-related genes and a terpene biosynthetic gene cluster showing similarity to carotenoid biosynthetic clusters. Based on the polyphasic taxonomic evidence, strain S9268^T^ represents a novel species within the genus *Pseudomonas*, for which the name *Pseudomonas yongnianensis* sp. nov. is proposed. The type strain is S9268^T^ (=ACCC 64339^T^ = JCM 38183^T^).

## 1. Introduction

The genus *Pseudomonas*, a member of the family Pseudomonadaceae within the class Gammaproteobacteria, is characterized by its remarkable metabolic plasticity and ecological adaptability [1,2]. Members of this genus are widely distributed across diverse habitats, ranging from soil [3] and aquatic [4] environments to animal- and plant-associated ecosystems [5,6], where they play critical roles in nutrient cycling, bioremediation, plant growth promotion, and in some cases, pathogenicity [7,8]. From a taxonomic perspective, the genus has experienced significant growth in recent years, now encompassing 384 species with validly published names, as documented in the List of Prokaryotic Names with Standing in Nomenclature (LPSN; https://lpsn.dsmz.de, accessed on 15 September 2026). [9]. This rapid increase reflects both the extensive genetic diversity within the genus and the continuous discovery of novel lineages from underexplored ecological niches.

Among the phenotypic traits observed within the genus, the production of diffusible fluorescent pigments under iron-limited conditions is a characteristic feature of a large subset of *Pseudomonas* species, commonly referred to as fluorescent pseudomonads [10,11,12]. These pigments, primarily pyoverdines, function as high-affinity siderophores that facilitate iron acquisition in competitive environments such as the rhizosphere [11]. Fluorescent pseudomonads are frequently isolated from plant rhizosphere and have been extensively studied for their capacity to promote plant growth through mechanisms such as phytohormone production, nutrient solubilization, and the biosynthesis of antimicrobial secondary metabolites [13,14]. Consequently, the rhizosphere of crops represents a promising reservoir for the discovery of novel fluorescent *Pseudomonas* strains with potential biotechnological applications.

The rhizosphere of high-yield agricultural fields represents a particularly dynamic and competitive microbial habitat, shaped by intensive agronomic practices, continuous root exudation, and complex plant–microbe–soil interactions [15,16]. In China, the North China Plain is one of the most productive wheat-growing regions, yet the diversity of root-associated *Pseudomonas* populations in these managed ecosystems remains incompletely characterized [17]. Systematic isolation and taxonomic characterization of novel fluorescent pseudomonads from such environments not only contribute to the fundamental understanding of bacterial diversity, but may also yield strains with beneficial traits applicable to sustainable agriculture.

During a survey of bacterial diversity in the rhizosphere of wheat grown in a high-yield field in Yongnian District, Handan City, Hebei Province, China, a fluorescent *Pseudomonas* strain, designated S9268^T^, was isolated. This study seeks to clarify the taxonomic status of strain S9268^T^ through a comprehensive polyphasic strategy encompassing: (i) phylogenetic reconstruction using both 16S rRNA gene sequences and multilocus sequence analysis (MLSA); (ii) genome-based metrics—specifically average nucleotide identity (ANI) and digital DNA–DNA hybridization (dDDH); (iii) detailed phenotypic assessment; and (iv) chemotaxonomic analysis, including cellular fatty acid and polar lipid profiling. In addition, the genome was analyzed for features potentially relevant to plant–microbe interactions, including the presence of a type III secretion system (T3SS) gene cluster and a terpene biosynthetic cluster putatively associated with carotenoid production. Based on the collective polyphasic evidence, strain S9268^T^ represents a novel species within the genus *Pseudomonas*, for which the name *Pseudomonas yongnianensis* sp. nov. is proposed.

## 2. Materials and Methods

### 2.1. Sample Collection and Bacterial Isolation

Strain S9268^T^ was isolated from the rhizosphere soil of wheat collected from a high-yield field in Yongnian District, Handan City, Hebei Province, China (36°43′48″ N, 114°41′24″ E) in May 2023. About 1 g of rhizosphere soil was thoroughly mixed with 9 mL of sterile distilled water and agitated at 200 rpm for 60 min at 28 °C. Tenfold serial dilutions were prepared using the same sterile diluent, and 100 μL of each dilution (ranging from 10^−1^ to 10^−5^) was evenly spread onto King’s B (KB) agar medium [18]. The KB formulation (per liter) consisted of 20 g proteose peptone No. 3 (Becton, Dickinson and Company, Franklin Lakes, NJ, USA), 1.5 g dipotassium hydrogen phosphate (K_2_HPO_4_) (Xilong Scientific Co., Ltd., Shantou, Guangdong, China), 1.5 g magnesium sulfate heptahydrate (MgSO_4_·7H_2_O) (Xilong Scientific Co., Ltd., Shantou, Guangdong, China), 10 mL glycerol (Tianjin Zhiyuan Chemical Reagent Co., Ltd., Tianjin, China), and 15 g agar. Colonies were allowed to develop under aerobic conditions at 28 °C for 24–48 h. Colonies exhibiting fluorescence under UV illumination (254 nm) using a UV lamp/transilluminator (Gongyi Yuhua Instrument Co., Ltd., Gongyi, Henan, China) were selected and purified by streaking on KB agar. The isolated bacterial strain was routinely grown in King’s B medium at 28 °C and stored long-term at −80 °C in a 20% (*v*/*v*) glycerol solution.

The type strains of phylogenetically proximal species—*Pseudomonas asturiensis* LMG 26898^T^, *Pseudomonas avellanae* LMG 21662^T^, and *Pseudomonas lijiangensis* JCM 35177^T^—were acquired from the Belgian Coordinated Collections of Microorganisms (BCCM/LMG, Ghent, Belgium) and the Japan Collection of Microorganisms (JCM, Tsukuba, Japan). To ensure comparability in phenotypic and chemotaxonomic evaluations, all reference strains were grown under identical culture conditions as strain S9268^T^.

### 2.2. Genome Sequencing, Assembly, and Annotation

Genomic DNA was isolated from strain S9268ᵀ using the E.Z.N.A.^®^ Bacterial DNA Kit (Omega Bio-Tek, Norcross, GA, USA), following the supplier’s recommended protocol. Whole-genome sequencing was carried out on an Illumina NextSeq 2000 system with paired-end reads of 2 × 150 bp. Raw reads were quality-filtered using Trimmomatic v0.33 and de novo assembled with SPAdes v3.6.2. The quality of the resulting assembly was assessed using CheckM v1.2.4 to ensure completeness higher than 95% and contamination below 5%.

Protein-coding genes were predicted using Prodigal v2.6.3. Transfer RNA (tRNA) genes were identified using tRNAscan-SE v2.0, and ribosomal RNA (rRNA) genes were predicted using cmsearch v1.1.3. Function of predicted proteins was annotated against the NCBI non-redundant (NR) database, the Evolutionary Genealogy of genes: Non-supervised Orthologous Groups (eggNOG) database v4.0 [19], the Kyoto Encyclopedia of Genes and Genomes (KEGG) database (kegg_201703) [20], and the Gene Ontology (GO) databases (releases20180910) [21].

The DDBJ database contains the draft genome sequence of strain S9268^T^, with accession numbers BAAMED010000001 to BAAMED010000020. The genome sequence of S9268^T^ can be found at https://www.ncbi.nlm.nih.gov/nuccore/3393630940 (accessed on 1 October 2026).

### 2.3. Genomic Relatedness Indices Analysis

To further clarify the taxonomic position of strain S9268^T^, overall genomic relatedness indices were accessed. In this study, FastANI v1.31 was used to calculate the average nucleotide identity (ANI) values, and Genome-to-Genome Distance Calculator (GGDC) version 3.0 (https://ggdc.dsmz.de/) (accessed on 12 February 2026) was employed to determine digital DNA–DNA hybridization (dDDH) values using formula 2, which is recommended for incomplete draft genomes [22]. The species delimitation criteria used were an ANI of 95–96% and a dDDH value of 70% [23,24].

### 2.4. 16S rRNA Gene and Multilocus Sequence Analysis (MLSA)-Based Phylogenetic Analysis

The 16S rRNA gene was amplified via PCR using the universal primers 27F (5′-AGAGTTTGATCCTGGCTCAG-3′) and 1492R (5′-TACGGYTACCTTGTTACGACTT-3′) [25]. Amplified fragments were purified and subjected to Sanger sequencing by GENEWIZ Ltd. (Suzhou, China). Taxonomic affiliation and nearest phylogenetic relatives were determined by comparing the obtained sequence against the EzBioCloud 16S rRNA database [26].

For MLSA, the partial sequences of four housekeeping genes—16S rRNA gene, *gyrB*, *rpoB*, and *rpoD*—were extracted from genomes of strain S9268^T^ and closely related type strains. Individual gene sequences were aligned using the ClustalW algorithm implemented in MEGA-X [27], manually trimmed to remove ambiguous positions, and concatenated in the following order: 16S rRNA gene (1497 bp), *gyrB* (2415 bp), *rpoB* (2259 bp), and *rpoD* (2167 bp), yielding a final alignment of 8338 bp.

Phylogenetic trees were inferred from both the 16S rRNA gene and a concatenated alignment of core housekeeping genes using the maximum-likelihood (ML) algorithm implemented in MEGA-X. Node robustness was assessed via bootstrap resampling with 1000 replicates. *Pseudomonas aeruginosa* ATCC 10145^T^ served as the outgroup for root placement.

### 2.5. Genomic Feature Mining

The genome of strain S9268^T^ was screened for features potentially relevant to plant–microbe interactions. Biosynthetic gene clusters (BGCs) responsible for secondary metabolite production were identified using antiSMASH v8.0 [28]. Protein secretion systems were identified using MacSyFinder v2.1.2 with the TXSScan module and default parameters, which includes the available models for type I–VI secretion systems (T1SS–T6SS) [29]. All predicted secretion system components were inspected manually. The presence of a type III secretion system (T3SS) gene cluster was further verified by blastp alignment of the predicted protein sequences against the NCBI nr database. The presence of a type III secretion system (T3SS) gene cluster was further verified by manual alignment of the predicted protein sequences against the NCBI NR database using blastp. Putative genes associated with plant growth promotion were detected by blastp analysis (e-value cutoff: ≤1 × 10^−5^; amino acid identity threshold: ≥40%) using reference sequences of 1-aminocyclopropane-1-carboxylate (ACC) deaminase, indole-3-acetamide hydrolase (IaaH), and diverse carbohydrate-active enzymes as query markers.

### 2.6. Physiological and Biochemical Characterization

Cellular morphology, dimensions, and flagellar configuration were visualized via transmission electron microscopy (TEM) using a Hitachi HT7700 instrument (Hitachi, Tokyo, Japan). Growth temperature range and optimum were determined in KB at 4, 10, 15, 20, 25, 28, 30, 35 and 37 °C for 24 h. The pH range for growth was assessed in KB broth at pH 6.0–9.0 (at 1.0 pH unit intervals) using the buffer system described by Xu et al. [30]. The impact of NaCl to growth was assessed in KB broth with NaCl concentrations from 1% to 4% (in 1% increments). All growth tests were performed in triplicate, and growth was monitored by measuring optical density at 600 nm (OD_600_) using a spectrophotometer.

Enzyme activities and further biochemical properties were tested with the API 20NE system (bioMérieux, Marcy-l’Etoile, France) in accordance with the supplier’s protocol. Profiles of carbon source utilization and chemical sensitivity were generated using Biolog GEN III MicroPlates (Biolog, Hayward, CA, USA). Prior to the API 20NE and Biolog assays, strain S9268^T^ and the reference strains were first cultivated on tryptic soy agar (TSA; BD Difco, Sparks, MD, USA) at 28 °C for 24 h. After inoculation, the API strips and Biolog plates were kept at 28 °C, with readings taken after 24–48 h in line with the corresponding manufacturer’s instructions.

### 2.7. Chemical Taxonomy Feature Analysis

For chemotaxonomic analyses, strain S9268^T^ and three closely related reference strains, *Pseudomonas asturiensis* LMG 26898^T^, *Pseudomonas avellanae* LMG 21662^T^, and *Pseudomonas lijiangensis* JCM 35177^T^, were cultured in tryptic soy broth (TSB; BD Difco) at 28 °C for 24 h on a rotary shaker (200 rpm). *Pseudomonas viridiflava* DSM 11124^T^, which was also identified as a closely related species based on ANI and dDDH values, was not included in the experimental phenotypic and chemotaxonomic analyses because of biosafety-related constraints on its acquisition and handling. Cells were harvested by centrifugation (8000× *g*, 10 min, 4 °C), washed twice with sterile distilled water, and freeze-dried.

Fatty acid composition was determined by the gas chromatographic analysis (Agilent 7890 BGC) of fatty acid methyl esters (FAMEs), following the standard operating procedure of the MIDI Sherlock Microbial Identification System and employing the RTSBA 6.1 database for peak assignment [31,32]. Polar lipid profiles were resolved and visualized via two-dimensional thin-layer chromatography (TLC) [33]. Respiratory quinones were extracted from lyophilized cells and quantified using reversed-phase high-performance liquid chromatography (HPLC) on a Shimadzu LC-20A system [34].

### 2.8. Assessment of Plant Growth-Promoting Traits

The plant growth-promoting (PGP) traits of strain S9268^T^ were evaluated in vitro using standard qualitative assays. All tests were performed in triplicate.

#### 2.8.1. Indole-3-Acetic Acid (IAA) Production

Indole-3-acetic acid (IAA) biosynthesis was quantified spectrophotometrically using the Salkowski assay [35]. Briefly, strain S9268^T^ was cultured in King’s B (KB) broth containing 0.8 g·L^−1^ L-tryptophan at 28 °C with shaking at 200 rpm for 96 h. Following centrifugation, 100 μL of the cell-free supernatant was reacted with an equal volume of Salkowski reagent; a pink-to-red chromogenic response, measurable at 530 nm, served as a qualitative indicator of IAA synthesis.

#### 2.8.2. Ammonia Production

Ammonia production was tested in Peptone Ammonification Medium after 48 h of incubation at 28 °C and 220 rpm. Nessler’s reagent was added to 200 μL culture; a yellow to brown-red precipitate indicated positive activity [36].

#### 2.8.3. Siderophore Production

Siderophore synthesis was evaluated using chrome azurol S (CAS) agar medium; a distinct orange-colored zone surrounding the colony following incubation at 28 °C for 72 h was interpreted as a positive signal for siderophore secretion [37].

#### 2.8.4. Extracellular Enzyme Activities

Cellulase, protease, and amylase activities were tested on CMC (Carboxymethylcellulose sodium Medium) agar, skim milk agar, and starch agar, respectively. Clear zones around colonies after 1–3 days at 28 °C indicated positive enzymatic activity [38].

#### 2.8.5. Phosphate and Potassium Solubilization

Inorganic and organic phosphate solubilization were tested on Pikovskaya’s agar and modified Pikovskaya’s agar with phytic acid, respectively. Potassium solubilization was assessed on Aleksandrov agar. Clear halos after 3–5 days indicated solubilization activity [39].

### 2.9. Whole-Genome-Based Phylogenomic Tree of P. syringae Species Complex

Genome sequences of strain S9268^T^ and 23 type strains of the *P. syringae* species complex were retrieved from NCBI [40]. Single-copy orthologous proteins were identified with OrthoFinder v2.5.4 and DIAMOND v0.9.14, aligned with MAFFT v7.471, and concatenated. A maximum-likelihood tree was inferred using FastTree v2.1.10 with SH-like local support and visualized in iTOL v7 [41,42,43,44,45].

## 3. Results

### 3.1. Phylogenetic and Genomic Analyses

The 16S rRNA gene sequence of strain S9268^T^ (1497 bp; Genbank accession number PZ120410.1) was determined and compared against the EzBioCloud database. Strain S9268^T^ showed the highest 16S rRNA gene sequence similarity to *Pseudomonas avellanae* LMG 21662^T^ (99.60%), followed by *Pseudomonas viridiflava* DSM 11124^T^ (97.95%), *Pseudomonas asturiensis* LMG 26898^T^ (97.87%), and *Pseudomonas lijiangensis* JCM 35177^T^ (97.53%). Despite the high 16S rRNA gene sequence similarity, genomic relatedness indices clearly differentiated strain S9268^T^ from its closest relatives. The pairwise ANI values between strain S9268^T^ and the reference strains ranged from 82.9% to 83.5%, and the dDDH values ranged from 24.7% to 25.6% (Table 1), all well below the widely accepted species delineation thresholds of 95–96% for ANI and 70% for dDDH.

**Figure 1:**
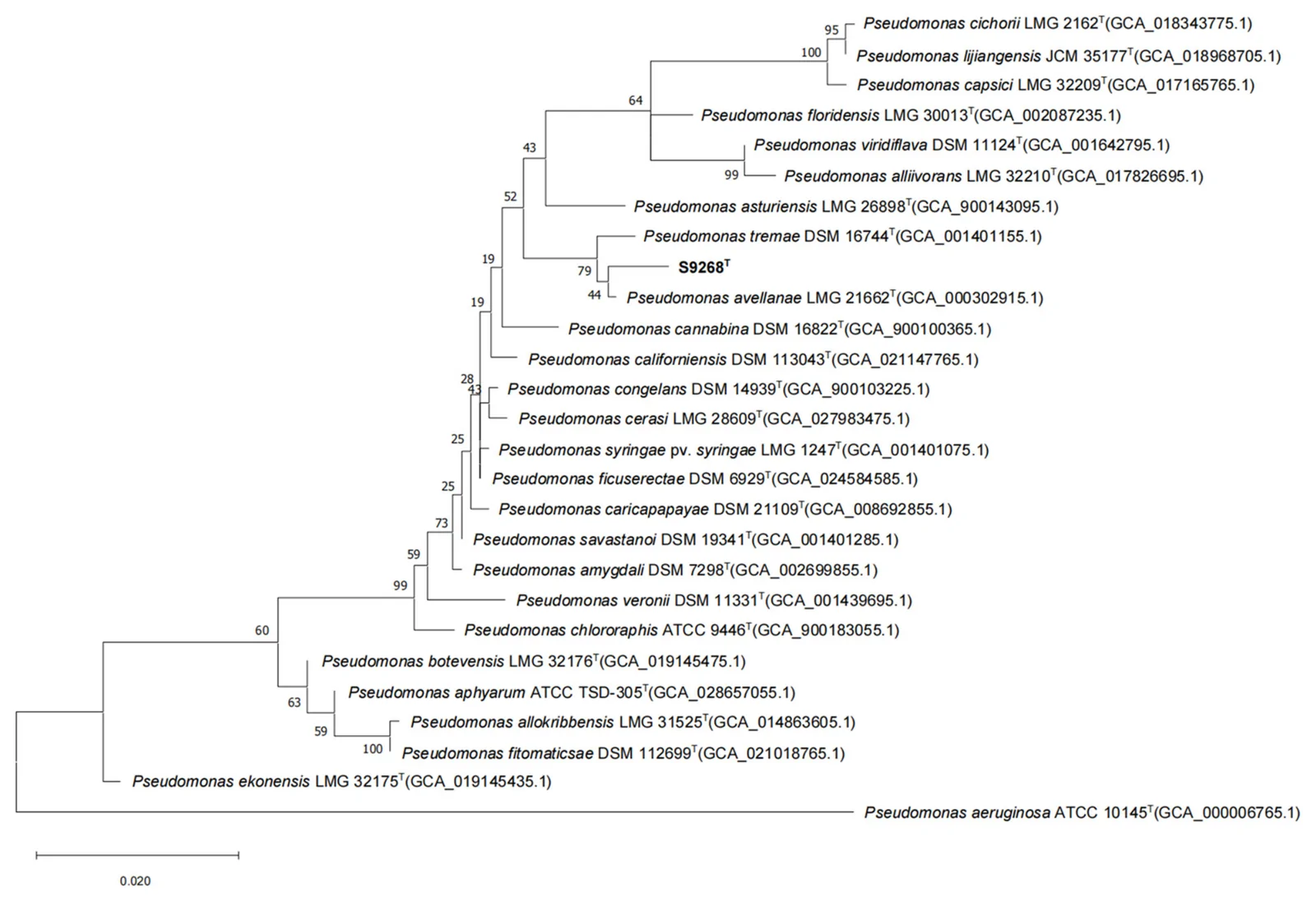
A maximum-likelihood phylogenetic tree was inferred from the 16S rRNA gene sequences of strain S9268^T^ and its phylogenetically closest relatives. Analyses were performed in MEGA-X using the Tamura–Nei model, with branch support assessed via 1000 bootstrap replicates; values ≥50% are displayed at corresponding nodes. The scale bar represents 0.05 nucleotide substitutions per site.

**Figure 2:**
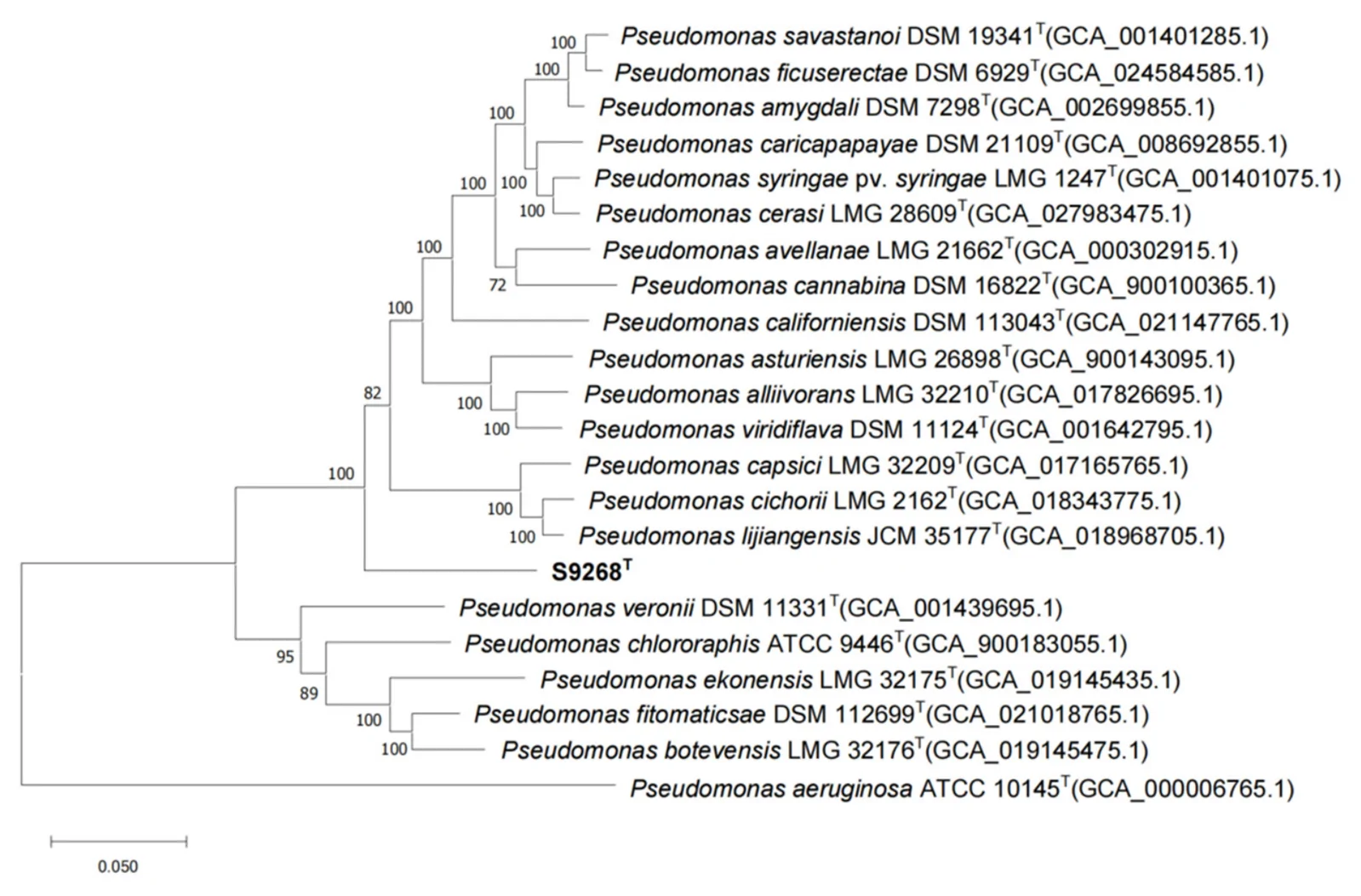
A maximum-likelihood phylogeny was reconstructed using concatenated nucleotide sequences of four core housekeeping genes—16S rRNA, *gyrB*, *rpoB*, and *rpoD*—to resolve the evolutionary relationships among strain S9268^T^ and its closest phylogenetic neighbors. The analysis was conducted in MEGA-X under the GTR + G + I model (selected by ModelFinder), with node support evaluated through 1000 bootstrap iterations; values ≥50% are indicated at relevant branches. The scale bar corresponds to 0.05 substitutions per site.

Phylogenetic analysis based on the 16S rRNA gene placed strain S9268^T^ within a clade together with *P. avellanae* and *P. tremae* (Figure 1). However, the branching pattern was not well-resolved, with strain S9268^T^ forming an independent lineage loosely associated with *P. avellanae* LMG 21662^T^ (bootstrap support of 44%). To achieve greater phylogenetic resolution, MLSA was performed using concatenated partial sequences of four housekeeping genes (16S rRNA gene, *gyrB*, *rpoB*, and *rpoD*; total alignment length 8338 bp) [46]. In the MLSA tree, strain S9268^T^ formed a distinct lineage with robust bootstrap support (100%), clearly separated from *P. asturiensis* LMG 26898^T^, *P. avellanae* LMG 21662^T^, and *P. lijiangensis* JCM 35177^T^ (Figure 2). This topology, combined with the ANI and dDDH values, unambiguously supports the recognition of strain S9268^T^ as a novel species within the genus *Pseudomonas*. Based on these results, *P. asturiensis* LMG 26898^T^, *P. avellanae* LMG 21662^T^, and *P. lijiangensis* JCM 35177^T^ were selected as reference strains for subsequent comparative phenotypic and chemotaxonomic analyses.

To further clarify the position of strain S9268^T^ within the currently established phylogenetic framework of the *P. syringae* species complex, a whole-genome phylogenetic tree was constructed using strain S9268^T^ and 23 type strains representing the recognized species of the complex (Figure S1). Strain S9268^T^ formed a distinct lineage within the large clade comprising *P. floridensis*, *P. alliivorans*, *P. viridiflava*, *P. triticifolii*, *P. asturiensis*, *P. tremae*, *P. cannabina*, *P. avellanae*, and related species, but it did not cluster with any known species. It was clearly separated from *P. syringae* sensu stricto [40]. These results indicate that strain S9268^T^ represents a novel species that is phylogenetically associated with, but distinct from, the currently recognized species of the *P. syringae* species complex.

### 3.2. Morphological, Physiological, and Biochemical Characteristics

Cells of strain S9268^T^ were Gram-negative, aerobic, rod-shaped (0.8–1.0 μm wide and 2.5–3.0 μm long), and motile by means of multiple polar flagella (Figure S2b). Colonies grown on TSA at 28 °C for 48 h were circular, smooth, convex, and yellowish. On KB agar, the colonies were pale yellow, circular, flat, mucoid (Figure S2a), and exhibited fluorescence under UV light (254 nm).

Growth of strain S9268^T^ occurred at temperatures ranging from 4 to 37 °C, with optimal growth at 28 °C.The pH range for growth was 6.0–9.0, with an optimum at pH 7.0. The strain grew in the presence of 1–4% (*w*/*v*) NaCl, with optimal growth observed at 1% (*w*/*v*) NaCl (Table S1). These growth characteristics are consistent with those of typical soil-dwelling, mesophilic *Pseudomonas* species [47,48].

Detailed physiological and biochemical profiles of strain S9268^T^ and the three reference strains are presented in Table 2. According to the API 20NE tests, strain S9268^T^ differed from the related type strains by several physiological characteristics. It was positive for d-mannose assimilation, whereas *Pseudomonas asturiensis* LMG 26898^T^, *Pseudomonas avellanae* LMG 21662^T^ and *Pseudomonas lijiangensis* JCM 35177^T^ showed weak reactions. Gelatin hydrolysis was negative in strain S9268^T^ but positive in *Pseudomonas asturiensis* LMG 26898^T^. Furthermore, strain S9268^T^ could assimilate l-arabinose, gluconate and decanoic acid, unlike *Pseudomonas avellanae* LMG 21662^T^, which showed negative reactions for these substrates. Biolog GEN III assays revealed several differential metabolic characteristics among strain S9268^T^ and the related type strains. Strain S9268^T^ was able to grow in the presence of 4% NaCl and utilize d-galactose, whereas *Pseudomonas asturiensis* LMG 26898^T^, *Pseudomonas avellanae* LMG 21662^T^ and *Pseudomonas lijiangensis* JCM 35177^T^ showed negative reactions for these characteristics. Notably, although d-mannose was tested in both the API 20NE and Biolog GEN III assays, the results differed slightly (positive in API 20NE vs. weak in Biolog GEN III). This difference likely reflects the distinct assay principles of the two systems—assimilation in minimal medium for API 20NE versus redox-based carbon source utilization for Biolog GEN III—and should not be regarded as a contradiction. In contrast, strain S9268^T^ was unable to utilize l-aspartic acid and quinic acid, while all three reference strains exhibited positive reactions. Strain S9268^T^ could be distinguished from *Pseudomonas asturiensis* LMG 26898^T^ by several carbon utilization patterns. *Pseudomonas asturiensis* LMG 26898^T^ was positive for d-fructose, myo-inositol, d-galacturonic acid, l-galactonic acid lactone and glucuronamide, whereas strain S9268^T^ and the other two reference strains were negative for these substrates. Compared with strain S9268^T^, *Pseudomonas avellanae* LMG 21662^T^ was positive for sucrose, d-mannitol and d-arabitol but negative for α-d-glucose, 1% sodium lactate and l-glutamic acid, whereas S9268^T^ and other two strains showed the opposite pattern. In comparison with *Pseudomonas lijiangensis* JCM 35177^T^, strain S9268^T^ differed in d-aspartic acid utilization, with strain S9268^T^ being negative and *Pseudomonas lijiangensis* JCM 35177^T^ being positive. These distinct metabolic profiles further differentiate strain S9268^T^ from its closely related type strains and support its classification as a novel species.

### 3.3. Chemotaxonomic Characterization

The cellular fatty acid profile of strain S9268^T^ was characterized by the predominance of C_16:0_ (25.56%), summed feature 3 (C_16:1_ ω7c and/or C_16:1_ ω6c; 24.04%), summed feature 8 (C_18:1_ ω7c and/or C_18:1_ ω6c; 14.96%), and C_17:0_ cyclo (11.76%) (Table 3). Hydroxylated fatty acids, including C_12:0_ 3-OH (5.25%), C_12:0_ 2-OH (4.40%) and C_10:0_ 3-OH (4.03%), were also detected. The overall fatty acid composition of strain S9268^T^ was consistent with those generally reported for members of the genus *Pseudomonas*. Comparative analysis of cellular fatty acid profiles revealed several differential characteristics between strain S9268^T^ and the related type strains. The proportion of summed feature 3 in strain S9268^T^ (24.04%) was lower than those in *Pseudomonas asturiensis* LMG 26898^T^ (46.52%), *Pseudomonas avellanae* LMG 21662^T^ (31.47%) and *Pseudomonas lijiangensis* JCM 35177^T^ (36.77%). In contrast, summed feature 8 was more abundant in strain S9268^T^ (14.96%) than in *P. asturiensis* LMG 26898^T^ (5.15%) and *P. avellanae* LMG 21662^T^ (10.32%), while it showed a comparable proportion to that of *P. lijiangensis* JCM 35177^T^ (13.04%). Notably, strain S9268^T^ lacked summed feature 7 (C_19:1_ ω7c and/or C_19:1_ ω6c and/or C_19:0_ cyclo ω7c), which was detected in all three reference strains (*P. asturiensis* LMG 26898^T^, *P. avellanae* LMG 21662^T^ and *P. li-jiangensis* JCM 35177^T^). In addition, C_17:0_ cyclo was present at a relatively high proportion in strain S9268^T^ (11.76%), whereas it was absent in *P. asturiensis* LMG 26898^T^ and *P. lijiangensis* JCM 35177^T^ and detected at a lower proportion in *P. avellanae* LMG 21662^T^ (6.49%). These distinctive cellular fatty acid characteristics further differentiate strain S9268^T^ from its closely related type strains.

Polar lipid profiling of strain S9268^T^ identified phosphatidylethanolamine (PE), diphosphatidylglycerol (DPG), phosphatidylglycerol (PG), three uncharacterized aminophospholipids (APL1–APL3), one unidentified phosphoglycolipid (PL), and one unknown polar lipid (L) (Figure S3). This composition aligns closely with the typical polar lipid pattern observed across *Pseudomonas* species. Ubiquinone-9 (Q-9) was the major respiratory quinone—consistent with the universal quinone signature of the genus *Pseudomonas*.

Taken together, the chemotaxonomic data confirm that strain S9268^T^ belongs to the genus *Pseudomonas*, while the distinctive fatty acid profile provides further evidence for its separation from phylogenetically related species.

### 3.4. Plant Growth-Promoting Traits

**Figure 3:**
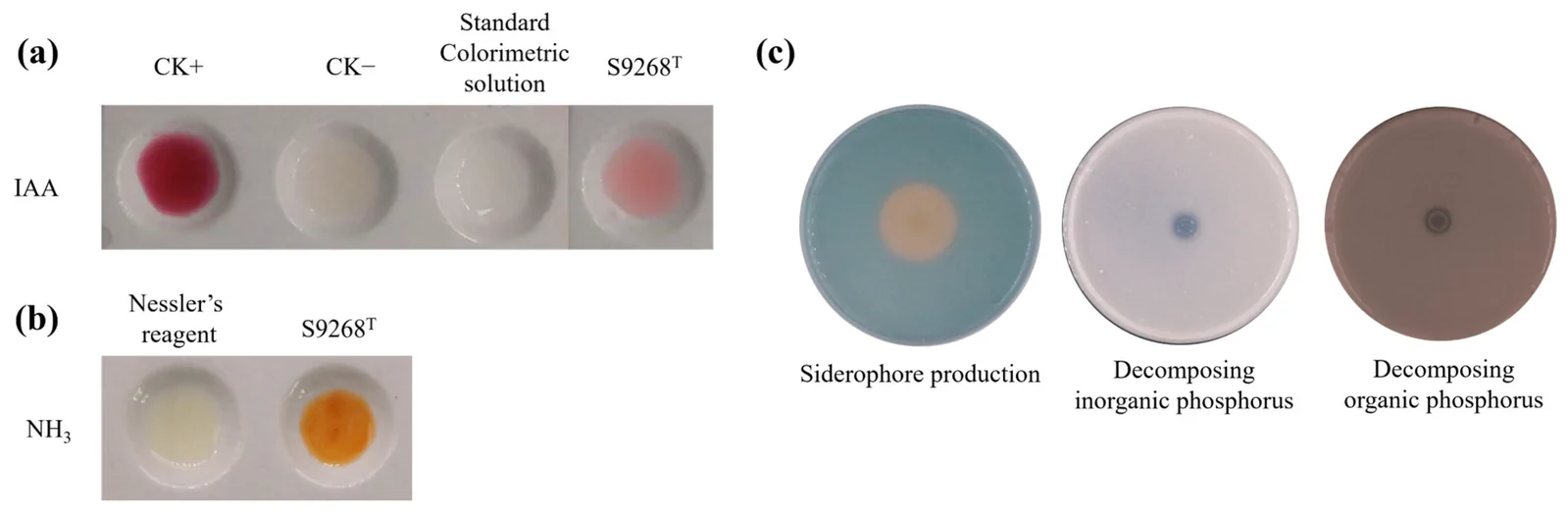
In vitro test of plant growth-promoting traits of strain S9268^T^. (**a**) IAA production, CK+ indicates that 100 µL of IAA solution (100 mg/L) was added to the colorimetric solution; CK− indicates that KB medium was added to the colorimetric solution; (**b**) ammonia production; (**c**) siderophore production, decomposing inorganic phosphorus, and decomposing organic phosphorus.

In vitro assays showed that strain S9268^T^ possessed various plant growth-promoting (PGP) traits. IAA production was verified by the appearance of a pink coloration following the addition of Salkowski reagent (Figure 3a). Ammonia production was demonstrated by the formation of a brown-red precipitate following the addition of Nessler’s reagent (Figure 3b). Siderophore production was observed on CAS agar, as evidenced by the formation of an orange halo surrounding the colony. Additionally, strain S9268^T^ exhibited phosphate-solubilizing activity against both inorganic and organic forms (Figure 3c). However, no cellulase, amylase, protease activity or potassium-solubilizing ability was detected under the tested conditions.

Strain S9268^T^ was isolated from the wheat rhizosphere from a high-yield farmland. During isolation, we specifically targeted fluorescent pseudomonads by screening for their ability to produce fluorescence, because fluorescent pseudomonads represent the *Pseudomonas* group most frequently associated with plant-growth-promoting bacteria and are considered a major reservoir of PGP bacteria within the genus *Pseudomonas*. These PGP-related traits suggest several potential roles of S9268^T^ in the wheat rhizosphere. Antibacterial activity may contribute to the suppression of plant pathogens; IAA production may influence root development and hormonal balance; ammonia production and phosphate solubilization may improve nitrogen and phosphorus availability; and siderophore production may enhance iron acquisition and contribute to competition with phytopathogens. Therefore, the novel species represented by strain S9268^T^ may have potential as a rhizosphere-associated PGP and/or biocontrol bacterium. As these are in vitro phenotypes and do not prove plant growth promotion functions, further inoculation and field experiments are needed to verify the actual PGP effects.

### 3.5. Genomic Characterization

#### 3.5.1. General Genome Features

The draft genome of strain S9268^T^ consisted of 5,250,102 bp assembled into 20 contigs, with a DNA G+C content of 61.2 mol% (Table 4). A total of 4693 protein-coding sequences (CDSs) were predicted, along with 55 tRNA genes and 7 rRNA genes. Functional annotation using the GO, KEGG and eggNOG databases revealed that the genome encodes a broad repertoire of genes involved in amino acid metabolism, carbohydrate metabolism, and membrane transport (Figures S4–S6), reflecting the metabolic versatility characteristic of the genus *Pseudomonas*.

#### 3.5.2. Biosynthetic Gene Clusters for Secondary Metabolites

AntiSMASH v8.0-based genome mining identified 10 candidate biosynthetic gene clusters (BGCs) associated with secondary metabolite production in the S9268^T^ genome (Figure S7). Among them, one terpene BGC (Region 1.3) showed high similarity to a carotenoid biosynthetic gene cluster, suggesting the potential for carotenoid production. This prediction is consistent with the yellowish pigmentation of colonies observed on TSA plates. In addition, a non-ribosomal peptide synthetase (NRPS)-type cluster (Region 1.1) showed low similarity to the pyoverdine biosynthetic gene cluster from *Pseudomonas protegens* Pf-5, indicating a possible role in siderophore production. One arylpolyene cluster (Region 2.1) with low similarity to APE Vf was also identified. Several other clusters were predicted without close matches to the characterized BGCs, including hydrogen cyanide-related clusters (Regions 1.2 and 4.1), a NAGGN cluster (Region 5.1), a redox cofactor-related cluster (Region 6.1), a terpene precursor cluster (Region 12.1), an NRPS-like cluster (Region 14.1), and an additional terpene cluster (Region 15.1). Collectively, the majority of predicted biosynthetic gene clusters (BGCs) exhibited minimal or no homology to currently annotated clusters in public databases, suggesting that strain S9268^T^ likely encodes a broad repertoire of secondary metabolite pathways—many of which could be previously uncharacterized. Nevertheless, functional confirmation of these BGCs necessitates targeted experimental approaches.

#### 3.5.3. Genomic Analysis of Secretion Systems and Plant-Associated Functional Genes

Secretion systems were predicted using the TXSScan module of MacSyFinder v2.1.2 against all available models (T1SS–T6SS) with default parameters, as described in Methods 2.5. Genomic analysis of secretion systems and selected functional genes or gene clusters revealed different distribution among strain S9268^T^ and the three closely related reference strains (Figure S7). Overall, the four strains share the universal presence of the genes of Type I, II, and V Secretion Systems (T1SS, T2SS, T5SS), as well as alginate biosynthesis, α-amylase, and diaminobutyrate. The most unique feature of strain S9268^T^ is the absence of ACC deaminase, while all three other strains (*P. lijianganensis* JCM 35177^T^, *P. avellanae* LMG21662^T^, and *P. asturiensis* LMG26898^T^) possess it. Compared to specific strains, S9268^T^ exhibits several differences. S9268^T^ possesses both T3SS and T6SS. However, T3SS is absent in *P. asturiensis* LMG26898^T^, and T6SS is absent in *P. avellanae* LMG21662^T^. Additionally, S9268^T^ contains the Psl polysaccharide synthesis cluster, sharing this trait only with *P. lijianganensis* JCM 35177^T^, while it is absent in *P. avellanae* LMG21662^T^ and *P. asturiensis* LMG26898^T^. These comparative genomic patterns indicate substantial variation in the repertoire of secretion systems and other putative functional determinants between strain S9268^T^ and its closely related species.

#### 3.5.4. Secretion Systems and Type III Secretion System Gene Cluster

Given the distinctive occurrence of T3SS-related genes in strain S9268^T^, their genomic organization was examined in greater detail. Rather than a canonical, complete Hrp-type T3SS locus, strain S9268^T^ possessed a highly streamlined T3SS-associated region containing conserved structural components of the Hrc apparatus (Figure S8). Unlike the well-characterized Hrp1-type T3SS locus of *Pseudomonas syringae* pv. *tomato* DC3000, strain S9268^T^ did not possess a complete and continuous T3SS pathogenicity island [49]. Instead, the identified region consisted of a compact core module comprising seven consecutively arranged structural genes, including *hrcV*, *hrcC*, *hrcN*, *hrcR*, *hrcS*, *hrcT* and *hrcU* (Figure S9). BLASTP version 2.5.0 analysis revealed significant homology between the predicted T3SS-related proteins of strain S9268^T^ and the corresponding components of the DC3000 T3SS system (Table S2). Among the core structural proteins, HrcN (GE000797) and HrcV (GE000807) showed high sequence conservation, with amino acid identities of 49.50% and 38.62% and query coverages of 95% and 98%, respectively (E-values < 1 × 10^−5^). HrcR (GE000794) also showed substantial similarity (43.63% identity, 98% query coverage). However, other structural components, including HrcC (GE000806) and HrcT (GE000792), displayed relatively low sequence identities (26.11% and 26.40%, respectively), suggesting considerable divergence among different components of the T3SS apparatus. Notably, the genomic organization of the S9268^T^ T3SS-associated region differed markedly from that of the DC3000 T3SS locus. In strain S9268^T^, the conserved *hrc* structural genes were arranged as a compact core cluster, whereas regulatory and accessory genes were not physically linked to this region. Homologs of key regulatory genes, including *hrpR* (GE001831), *hrpS* (GE001905) and *hrpL* (GE001865), were located at separate genomic positions, with amino acid identities of 48.73%, 41.40% and 27.56%, respectively. Additional T3SS-related proteins, including ORF1 (GE003569), ORF6 (GE002175) and ORF11 (GE000837), were also identified at dispersed genomic locations.

Together, these findings indicate that strain S9268^T^ harbors a highly streamlined and reorganized T3SS-related system rather than a canonical DC3000-like complete T3SS gene cluster. The conserved core structural genes suggest the retention of essential T3SS-related components, whereas the divergence and genomic separation of accessory and regulatory elements indicate extensive evolutionary remodeling of this secretion system. Although strain S9268^T^ harbours a putative T3SS-associated region, this region is streamlined and reorganised rather than a canonical complete Hrp-type T3SS pathogenicity island. The core *hrc* genes are arranged in a compact cluster, whereas key regulatory genes such as *hrpR*, *hrpS*, and *hrpL* are located at separate genomic positions and show relatively low sequence identity to their counterparts in *P. syringae* pv. tomato DC3000. Therefore, the presence of T3SS-related genes alone does not demonstrate that strain S9268^T^ is pathogenic. However, strain S9268^T^ is phylogenetically close to *P. viridiflava*, a recognized plant-pathogenic species, and its T3SS-associated region may still contribute to plant interactions. Consequently, the potential pathogenicity of strain S9268^T^ cannot be excluded based on genomic data alone. Before this strain is considered for agricultural application as a plant growth-promoting bacterium, its safety should be evaluated through a hypersensitive response assay on a non-host plant, pathogenicity tests on susceptible crop species, and determination of its host range. Such assessments will be essential to distinguish beneficial plant-associated traits from potential risks.

### 3.6. Description of Pseudomonas yongnianensis sp. nov.

*Pseudomonas yongnianensis* (yong.ni.an.en’sis. N.L. fem. adj. *yongnianensis*, pertaining to Yongnian District, Handan City, Hebei Province, PR China, the geographical origin of the type strain).

Cells are Gram-negative, aerobic, rod-shaped (0.8–1.0 μm in width and 2.5–3.0 μm in length), and motile by means of multiple polar flagella. Endospores are not produced. Colonies grown on Tryptic Soy Agar (TSA) at 28 °C for 48 h are circular, smooth, convex, and yellowish.On KB agar, the colonies were pale yellow, circular, flat, mucoid. Fluorescence is observed on KB agar under UV illumination (254 nm). Growth occurs at 4–37 °C (optimum, 28 °C), at pH 6.0–9.0 (optimum, pH 7.0), and in the presence of 1–4% (*w*/*v*) NaCl (optimum, 1%). In API 20NE tests, esculin is hydrolysed, but gelatin is not liquefied, and d-glucose, l-arabinose, d-mannose, d-mannitol, gluconate and decanoic acid are assimilated. In Biolog GEN III assays, α-d-glucose, d-galactose, d-fucose, d-mannose, d-gluconic acid, l-glutamic acid, and 1% sodium lactate are utilized as sole carbon sources, whereas sucrose, d-fructose, d-mannitol, d-arabitol, myo-inositol, d-galacturonic acid, l-galactonic acid lactone, glucuronamide, d-aspartic acid, l-aspartic acid, γ-aminobutyric acid, and quinic acid are not utilized. Growth occurs in the presence of 4% NaCl. The major cellular fatty acids (>10% of the total) are C_16:0_, summed feature 3 (C_16:1_ ω7c and/or C_16:1_ ω6c), summed feature 8 (C_18:1_ ω7c and/or C_18:1_ ω6c), and C_17:0_ cyclo. Hydroxylated fatty acids C_12:0_ 3-OH, C_12:0_ 2-OH, and C_10:0_ 3-OH are also present. The polar lipid profile consists of phosphatidylethanolamine (PE), diphosphatidylglycerol (DPG), phosphatidylglycerol (PG), three unidentified aminophospholipids (APL1–3), an unidentified phosphoglycolipid (PL), and an unidentified polar lipid (L). The major respiratory quinone is ubiquinone Q-9. The DNA G+C content of the type strain is 61.2 mol% (determined from the genome sequence). The draft genome sequence contains T3SS-related genes and a terpene biosynthetic gene cluster showing high similarity to carotenoid biosynthetic clusters.

The type strain, S9268^T^ (=ACCC 64339^T^ = JCM 38183^T^), was isolated from the rhizosphere soil of wheat in a high-yield field in Yongnian District, Handan City, Hebei Province, PR China (36°43′48″ N, 114°41′24″ E). The genome sequence of strain S9268^T^ has been deposited in the DDBJ database under accession numbers BAAMED010000001 to BAAMED010000020.

## 4. Conclusions

In this study, strain S9268^T^, isolated from the rhizosphere soil of wheat in a high-yield field in Yongnian, Hebei Province, China, was characterized using a polyphasic taxonomic approach. Phylogenetic analyses based on the 16S rRNA gene and multilocus sequence analysis (MLSA) placed strain S9268^T^ within the genus *Pseudomonas* but as a distinct lineage separated from its closest related species, including *Pseudomonas asturiensis*, *Pseudomonas avellanae*, and *Pseudomonas lijiangensis*. Genome-based comparisons, including ANI and dDDH analyses, revealed values below the accepted thresholds for species delineation, supporting the recognition of strain S9268^T^ as representing a novel species. Phenotypic and chemotaxonomic analyses further distinguished strain S9268^T^ from its related type strains, including its unique fatty acid profile characterized by a relatively high proportion of C_17:0_ cyclo and its ability to grow in the presence of 4% NaCl. Genome analysis revealed a streamlined T3SS-related gene cluster and a terpene biosynthetic gene cluster showing high similarity to carotenoid biosynthetic clusters, indicating diverse genomic features associated with secretion and secondary metabolism. In addition, strain S9268^T^ exhibited several plant growth-promoting traits in vitro, including indole-3-acetic acid production, siderophore production, ammonia production, and phosphate solubilization, suggesting its potential ecological significance as a plant-associated bacterium. However, because strain S9268^T^ carries a putative T3SS-associated region and is phylogenetically close to the plant-pathogenic species *P. viridiflava*, its safety to crops should be carefully evaluated before any agricultural application. Based on the combined phylogenetic, genomic, phenotypic, and chemotaxonomic evidence, strain S9268^T^ represents a novel species of the genus *Pseudomonas*, for which the name *Pseudomonas yongnianensis* sp. nov. is proposed.

## Figures and Tables

**Table 1:** ANI and dDDH values of S9268^T^ with the most closely related species.

Reference Strain	S9268^T^	
	ANI Values (%)	dDDH Values (%) [95% CI] *
*Pseudomonas lijiangensis* JCM 35177^T^ (GCA_018968705.1)	83.5	25.2 [22.9–27.7]
*Pseudomonas avellanae* LMG 21662^T^ (GCA_000302915.1)	83.1	25.6 [23.3–28.1]
*Pseudomonas viridiflava* DSM 11124^T^ (GCA_001642795.1)	83.4	25.1 [22.8–27.6]
*Pseudomonas asturiensis* LMG 26898^T^ (GCA_900143095.1)	83.4	24.9 [22.6–27.4]
*Pseudomonas floridensis* LMG 30013^T^ (GCA_002087235.1)	83.3	24.9 [22.6–27.4]
*Pseudomonas cichorii* LMG 2162^T^ (GCA_018343775.1)	83.3	24.8 [22.5–27.3]
*Pseudomonas capsici* LMG 32209^T^ (GCA_017165765.1)	83.2	24.9 [22.6–27.4]
*Pseudomonas alliivorans* LMG 32210^T^ (GCA_017826695.1)	83.3	24.7 [22.4–27.2]
*Pseudomonas cerasi* LMG 28609^T^ (GCA_027983475.1)	83.0	25.0 [22.7–27.5]
*Pseudomonas congelans* DSM 14939^T^ (GCA_900103225.1)	83.0	25.0 [22.7–27.5]
*Pseudomonas cannabina* DSM 16822^T^ (GCA_900100365.1)	82.9	25.1 [22.8–27.6]
*Pseudomonas syringae* pv. *syringae* LMG 1247^T^ (GCA_001401075.1)	83.0	25.0 [22.7–27.5]

* Confidence interval, CI.

**Table 2:** Differential physiological and biochemical characteristics of strain S9268^T^ and closely related species.

Characteristic	1	2	3	4
API20NE assay:				
gelatin	−	+	−	−
esculin	+	+	−	+
l-arabinose	+	+	−	+
d-mannose	+	w	w	w
decanoic acid	+	+	−	+
gluconate	+	+	−	+
d-glucose	+	+	w	+
Biolog GEN III assay:				
sucrose	−	−	+	−
4% NaCl	+	−	−	−
α-d-Glucose	+	+	−	+
d-malic acid	−	+	+	−
d-fructose	−	+	−	−
l-aspartic acid	−	+	+	+
d-fucose	w	+	−	−
1% sodium Lactate	+	+	−	+
d-mannitol	−	−	+	−
d-glucuronic acid	−	+	−	−
myo-inositol	−	+	w	−
d-fructose-6-PO4	−	w	−	−
d-aspartic acid	−	−	−	+
troleandomycin	−	w	−	−
γ-amino-butryric acid	−	+	+	−
l-glutamic acid	+	+	−	+
l-lactic acid	−	+	−	+
l-galactonic acid lactone	−	+	−	−
d-gluconic acid	+	+	−	−
d-mannose	w	−	−	−
glucuronamide	−	+	−	+
bromo-succinic acid	−	+	+	−
quinic acid	−	+	+	+
d-arabitol	−	−	+	−
α-keto-glutaric acid	+	+	−	+
mucic acid	+	+	−	+
d-galacturonic acid	−	+	−	−
d-galactose	+	−	−	−
acetic acid	+	w	+	−

Strains: 1, S9268^T^; 2, *Pseudomonas asturiensis* LMG26898^T^; 3, *Pseudomonas avellanae* LMG21662^T^; 4, *Pseudomonas lijiangensis* JCM 35177^T^; +, Positive; −, negative; w, weak.

**Table 3:** Fatty acid profile of strain S9268^T^ compared with that of the type strains from phylogenetically related *Pseudomonas* species.

Fatty Acid	1	2	3	4
Summed features:				
3 (C_16:1_ ω7c/ω6c)	24.04	46.52	31.47	36.77
7 (C_19:1_ ω7c/C_19:1_ ω6c/C_19:0_ cyclo ω7c)	-	2.02	1.6	1.88
8 (C_18:1_ ω7c/ω6c)	14.96	5.15	10.32	13.04
Saturated:				
C^12:0^	7.32	6.92	6.7	5.97
C^16:0^	25.56	21.91	26.76	26.28
Hydroxy:				
C_10:0_ 3-OH	4.03	5.54	4.05	4.04
C_12:0_ 2-OH	4.4	4.78	3.94	3.65
C_12:0_ 3-OH	5.25	4.54	4.82	4.79
Cyclopropane:				
C_17:0_ cyclo	11.76	-	6.49	-

Strains:1, S9268^T^; 2, *Pseudomonas asturiensis* LMG26898^T^; 3, *Pseudomonas avellanae* LMG21662^T^; 4, *Pseudomonas lijiangensis* JCM 35177^T^; -, not detected. All numerical values in this table are expressed as percentages (%).

**Table 4:** General genomic features of the S9268^T^.

Features	Chromosome S9268^T^
Size (bp)	5,250,102
rRNA genes	7
tRNAs	55
Average CDS length (nt)	993
Total CDSs size (bp)	4,662,144
Contigs	20
G+C content (%)	61.20
Number of total CDSs	4693

## Data Availability

The genome sequence of S9268^T^ is publicly available in the DDBJ database under the accession numbers BAAMED010000001 to BAAMED010000020. Direct links: https://www.ncbi.nlm.nih.gov/nuccore/3393630940 (accessed on 1 October 2026). The 16S rRNA gene sequence of strain S9268^T^ is publicly available in the Genbank database under the accession number PZ120410.1. Additional data supporting the findings of this study are available within the article.

## Supplementary Materials

The following supporting information can be downloaded at: https://www.mdpi.com/article/10.3390/microorganisms14102363/s1. Figure S1: Whole-genome-based phylogenomic tree based on single-copy orthologous proteins showing the phylogenetic relationships among strain S9268T and 23 type strains representing the currently recognized species of the *Pseudomonas syringae* species complex. The tree was constructed using OrthoFinder and Fasttree. Figure S2: Morphological and cultural characteristics of strain S9268T. Figure S3: Two-dimensional TLC plate of polar lipids extracted from S9268T and analysis of respiratory quinone by HPLC. Figure S4: Statistical chart of GO function annotation categories. Figure S5: KEGG annotation classification statistics. Figure S6: Statistical chart of functional gene classification of eggNOG. Figure S7: Putative BGCs in the S9268T genome predicted by antiSMASH. Figure S8: Selected biosynthetic, secretion system-related and catabolic genes or gene clusters were predicted using the genome sequences of strain S9268^T^ and the related species. Figure S9: Genetic organization of the type III secretion system gene cluster in strain S9268^T^. Table S1: Quantitative growth data for strain S9268^T^ under different temperature, pH, and NaCl conditions. Table S2: BLASTP-based homology analysis of T3SS components between strain S9268T and *Pseudomonas syringae* pv. *tomato* DC3000.
