# Supplementary material for: Pseudomonas yongnianensis sp. nov., Isolated from the Rhizosphere Soil of Wheat in a High-Yield Farmland
Source: Microorganisms. 2026 Oct 15;14(10):2363. doi: 10.3390/microorganisms14102363 (PMC13707685; doi:10.3390/microorganisms14102363)
Supplement: Supplementary file 1 [file microorganisms-14-02363-s001.zip › microorganisms-4607403-supplementary.pdf]

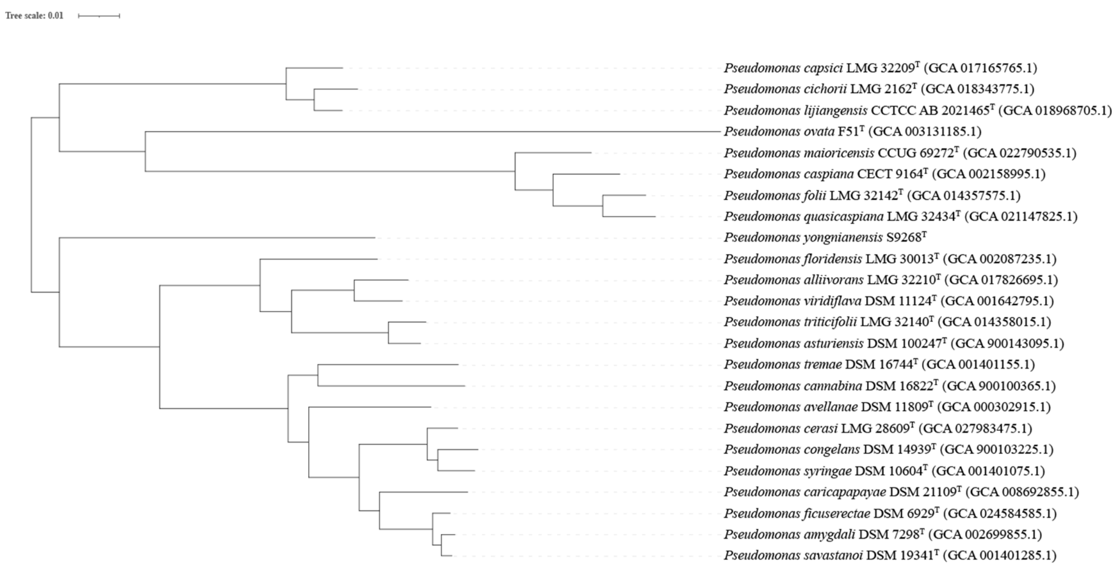

Figure S1. Whole-genome-based phylogenomic tree based on single-copy orthologous proteins showing the phylogenetic relationships among strain S9268^T and 23 type strains representing the currently recognized species of the *Pseudomonas syringae* species complex. The tree was constructed using OrthoFinder and Fasttree.

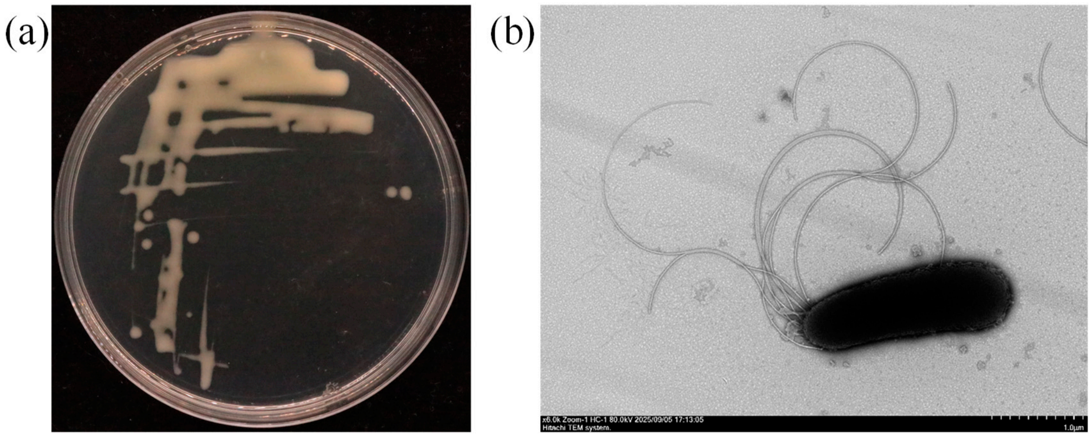

Figure S2. Morphological and cultural characteristics of strain S9268^{T}. (a) Colonies grown on King's B (KB) agar at 28°C for 48 h. (b) Transmission electron micrograph showing rod-shaped cells with multiple polar flagella. Bar, 2 μm.

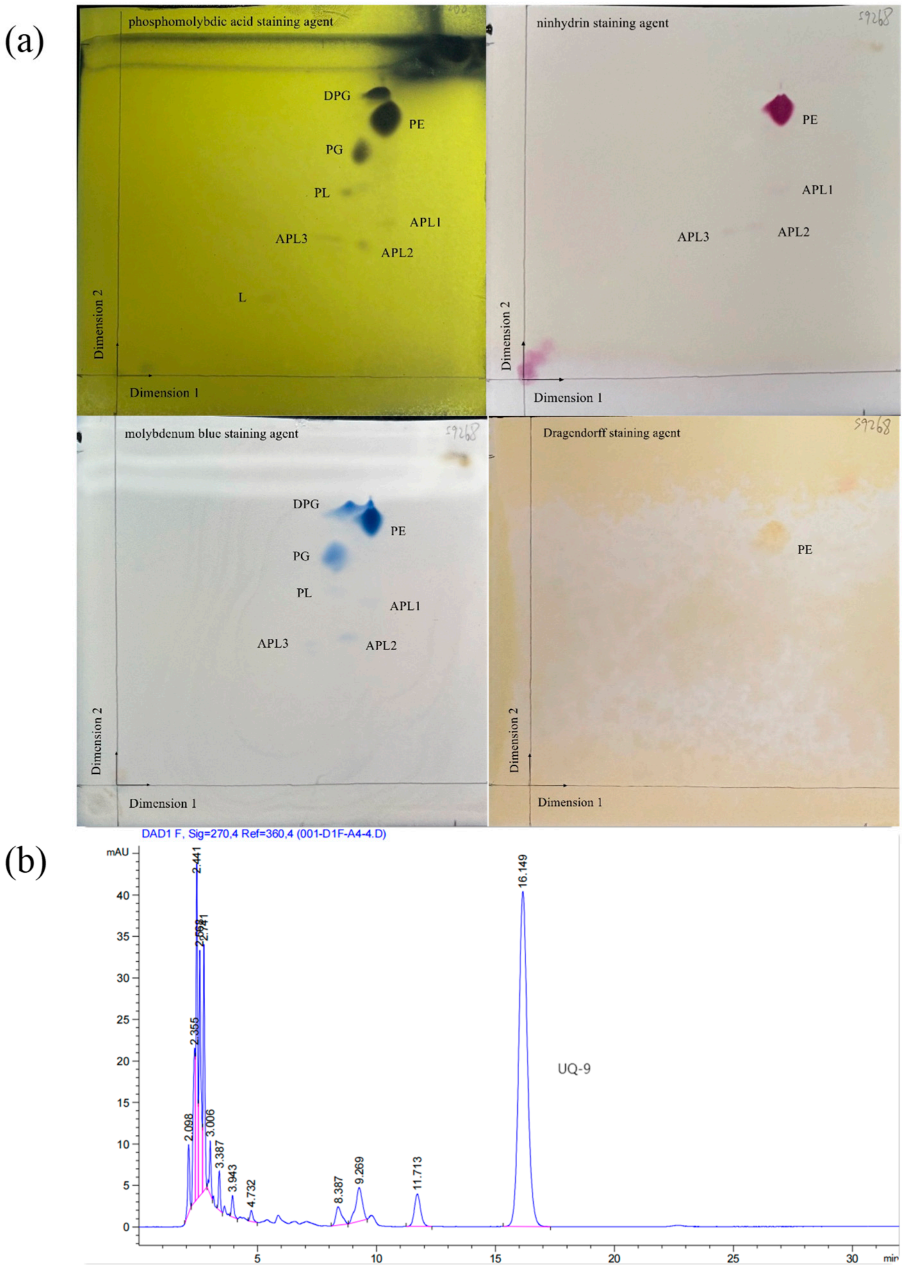

Figure S3. Two-dimensional TLC plate of polar lipids extracted from S9268^T and analysis of respiratory quinone by HPLC. (a): The plate was sprayed with 10% (v/v) molybdophosphoric acid to show all polar lipids present. DPG, diphosphatidylglycerol; PG, phosphatidylglycerol; PE, phosphatidylethanolamine; PL, unidentified phosphoglycolipids; APL, aminophospholipids; L, unidentified polar lipids. (b): the HPLC result of respiratory quinone.

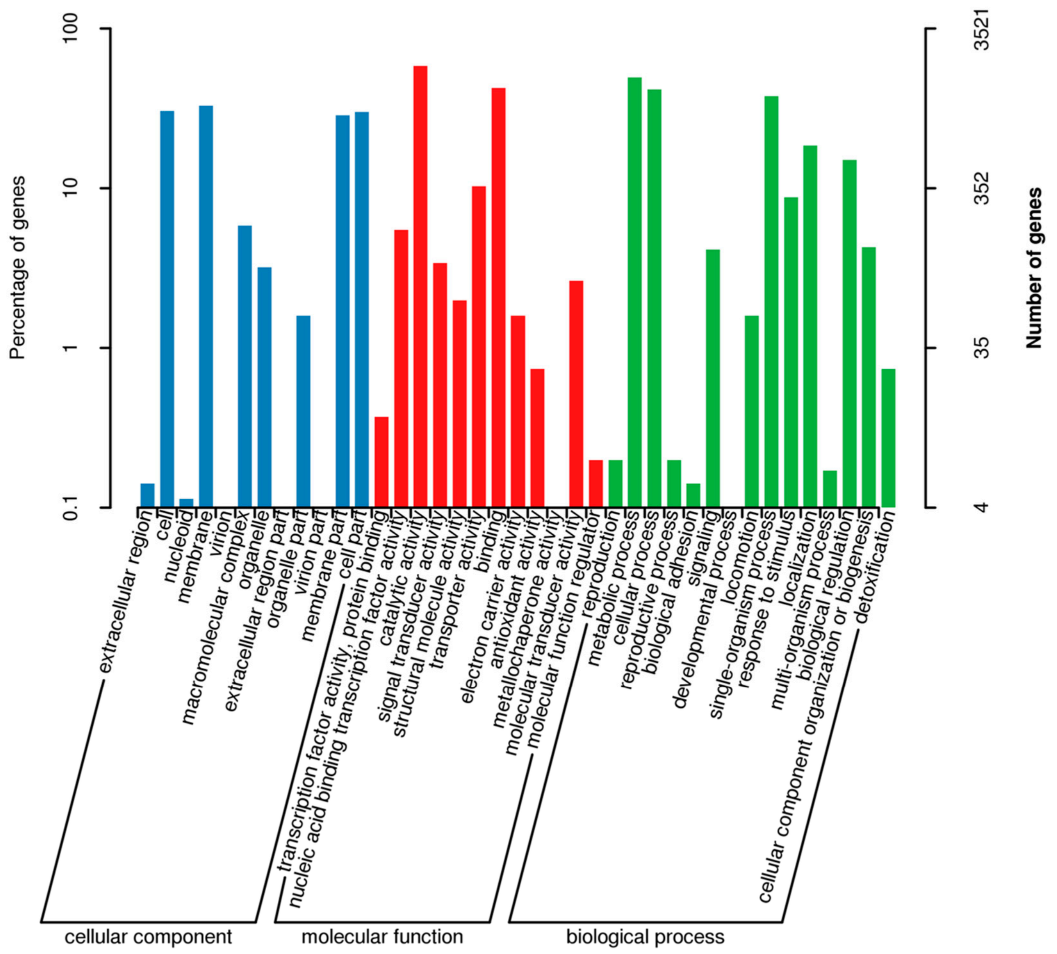

Figure S4. Statistical chart of GO function annotation categories.

The horizontal axis represents the content of each GO category, and the vertical axis shows the percentage of genes represented on the left and the total number of genes on the right. This figure shows the gene enrichment of each secondary function of GO in the context of all genes, reflecting the status of each secondary function in this context.

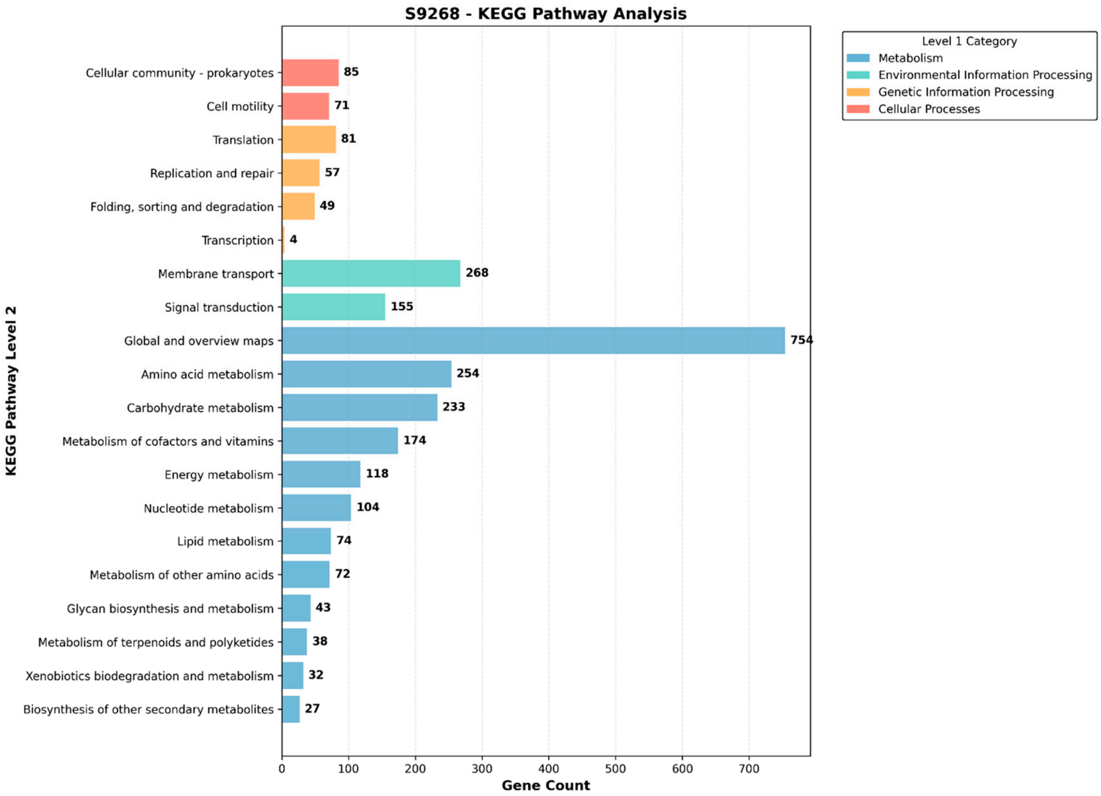

Figure S5. KEGG annotation classification statistics.

The vertical axis represents KEGG secondary classification, and the horizontal axis represents the number of genes.

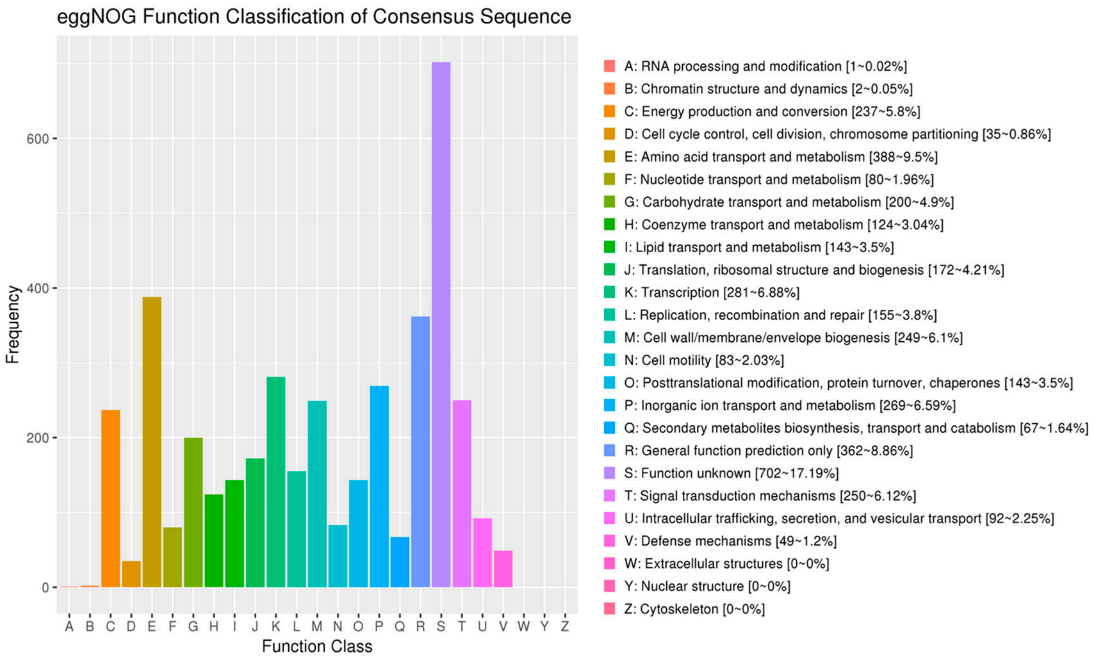

Figure S6. Statistical chart of functional gene classification of eggNOG.

The horizontal axis represents the categories of eggNOG, and the vertical axis represents the relative content of the corresponding functional genes.

antiSMASH version 8.0.4

Select genomic region:

Overview 1.1 1.2 1.3 2.1 4.1 5.1 6.1 12.1 14.1 15.1

Identified secondary metabolite regions using strictness 'relaxed'

Region	Type	From	To	Similarity Confidence	Most similar known cluster	
Region 1.1	NRP-metallophore, NRPS	1	92,353	Low	Pf-5 pyoverdine	NRPS:Type I
Region 1.2	hydrogen-cyanide	401,316	414,178			
Region 1.3	terpene	755,736	782,835	High	carotenoid	terpene
Region 2.1	arylpolyene	260,824	304,423	Low	APE Vf	other:other
Region 4.1	hydrogen-cyanide	1,549	15,572			
Region 5.1	NAGGN	372,221	387,043			
Region 6.1	redox-cofactor	229,746	251,938			
Region 12.1	terpene-precursor	80,269	101,156			
Region 14.1	NRPS-like	123,830	148,278			
Region 15.1	terpene	56,506	78,731			

Figure S7. Putative BGCs in the S9268^T genome predicted by antiSMASH.

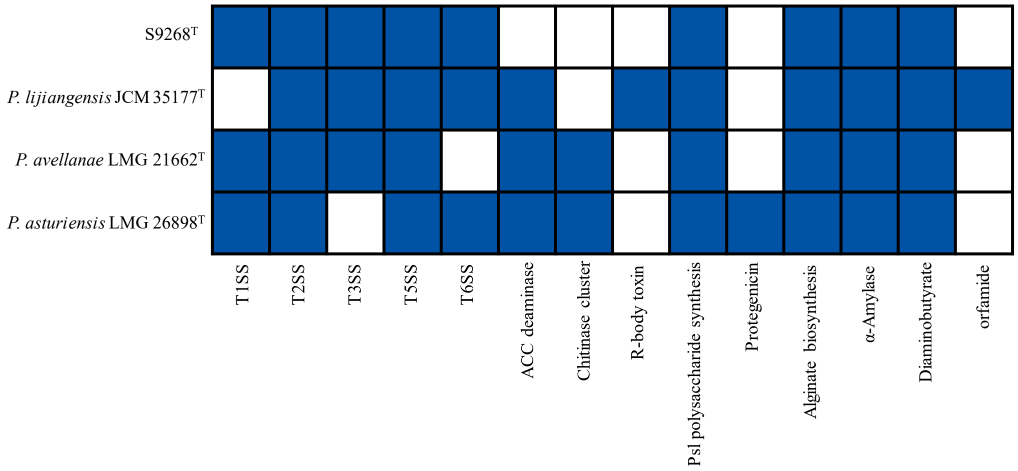

Figure S8. Selected biosynthetic, secretion system-related and catabolic genes or gene clusters were predicted using the genome sequences of strain S9268^{T} and the related species.

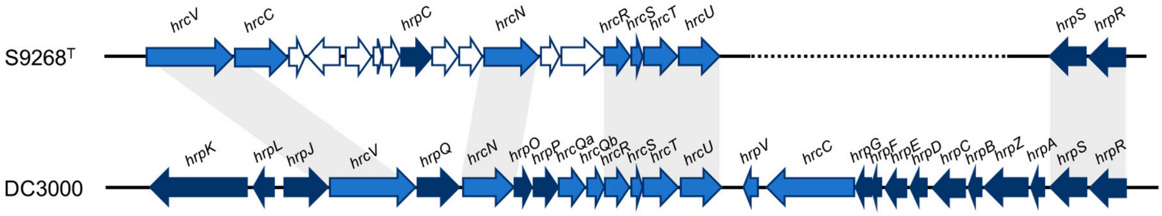

Figure S9. Genetic organization of the type III secretion system gene cluster in strain S9268^{T}.

Table S1. Quantitative growth data for strain S9268[T] under different temperature, pH, and NaCl conditions.

Condition	OD_{600} at 6 h	OD_{600} at 12 h	OD_{600} at 18 h	OD_{600} at 24 h
Temperature:				
4℃	0.030±0.006	0.151±0.008	0.212±0.013	0.260±0.035
10℃	0.202±0.008	0.370±0.009	0.587±0.004	0.900±0.014
15℃	0.815±0.037	1.262±0.022	1.638±0.019	1.899±0.009
20℃	0.720±0.086	1.471±0.036	1.795±0.012	1.948±0.004
25℃	0.933±0.052	1.209±0.006	1.944±0.008	2.010±0.006
28℃	0.927±0.014	1.243±0.036	1.957±0.019	2.191±0.030
30℃	1.545±0.031	1.870±0.016	1.898±0.009	1.912±0.013
35℃	0.847±0.014	1.329±0.005	1.532±0.025	1.844±0.012
37℃	0.540±0.078	0.808±0.023	1.274±0.027	1.536±0.060
NaCl:				
1%	−	−	−	2.172±0.467
2%	−	−	−	1.807±0.584
3%	−	−	−	1.817±0.124
4%	−	−	−	0.377±0.075
pH:				
6	−	−	−	1.665±0.023
7	−	−	−	1.918±0.006
8	−	−	−	0.807±0.033
9	−	−	−	0.017±0.002

Note: Cultures were grown in King's B (KB) broth. Growth was monitored by measuring OD_{600}. Values are shown as mean ± standard deviation of three replicates. −, below the detection threshold of the spectrophotometer .

Table S2. BLASTP-based homology analysis of T3SS components between strain S9268[T] and *Pseudomonas syringae* pv. *tomato* DC3000.

DC3000	S9268 Gene ID	Per. Ident (%)	Query Cover (%)	E value
		**Core genes**		
hrcC	GE000806	26.11	32	1.26e-08
hrcV	GE000807	38.62	98	8.51e-132
hrcN	GE000797	49.50	95	5.20e-119
hrcR	GE000794	43.63	98	1.04e-58
hrcS	GE000793	56.82	50	2.95e-10
hrcT	GE000792	26.40	94	1.81e-18
hrcU	GE004164	31.97	78	2.13e-27
		**Regulatory and auxiliary genes**		
hrpS	GE001905	41.40	94	1.60e-73
hrpR	GE001831	48.73	87	2.08e-80
hrpL	GE001865	27.56	81	2.05e-11
hrpK	GE002268	26.34	52	2.06e-29
hrpC	GE000800	35.39	90	8.10e-37
		**T3SS-related functional proteins**		
ORF1	GE003569	59.71	55	1.55e-112
ORF6	GE002175	77.67	84	2.45e-180
ORF11	GE000837	53.66	89	2.25e-44
gstA	GE002994	31.31	92	4.38e-20
